# Local photoreceptor cell death differences in the murine model of retinal detachment

**DOI:** 10.1038/s41598-021-97947-4

**Published:** 2021-09-22

**Authors:** Daniel E. Maidana, Lucia Gonzalez-Buendia, Joan W. Miller, Demetrios G. Vavvas

**Affiliations:** 1 Retina Service, Angiogenesis Lab, Massachusetts Eye and Ear Infirmary, Harvard Medical School, 243 Charles Street, Boston, MA 02114 USA; 2 Department of Ophthalmology and Visual Sciences, University of Illinois at Chicago, Chicago, IL USA

**Keywords:** Medical research, Cell biology, Cell death

## Abstract

To investigate local cell death differences in the attached and detached retina at different regions in a murine experimental retinal detachment model. Subretinal injection of sodium hyaluronate was performed in eight-week-old C57BL/6J mice. Retinal regions of interest were defined in relation to their distance from the peak of the retinal detachment, as follows: (1) attached central; (2) attached paracentral; (3) detached apex; and (4) detached base. At day 0, the outer nuclear layer cell count for the attached central, attached paracentral, detached apex, and detached base was 1247.60 ± 64.62, 1157.80 ± 163.33, 1264.00 ± 150.7, and 1013.80 ± 67.16 cells, respectively. There were significant differences between the detached base vs*.* attached central, and between detached base vs. detached apex at day 0. The detached apex region displayed a significant and progressive cell count reduction from day 0 to 14. In contrast, the detached base region did not show progressive retinal degeneration in this model. Moreover, only the detached apex region had a significant and progressive cell death rate compared to baseline. Immediate confounding changes with dramatic differences in cell death rates are present across regions of the detached retina. We speculate that mechanical and regional differences in the bullous detached retina can modify the rate of cell death in this model.

## Introduction

The separation of the neurosensory retina from the retinal pigment epithelium (RPE), or retinal detachment (RD), decreases the survival and alters the function of photoreceptors^1–3^. As the subretinal space is filled with fluid, debris, and infiltrating cells, and the gap between the outer retina and the choriocapillaris is increased, there is a decline in the availability of nutrients needed by the photoreceptors. This shortage, in context with the metabolic demands and fragility of photoreceptors, culminates in rod and cone cell death, which can occur as early as 12 hours post-detachment^4^. It has been previously demonstrated in a feline retinal detachment model, that the oxygen profile in the detached retina shares some features with the attached retina. However, the presence of a subretinal space increases the latency of oxygen delivery to the photoreceptors from the choriocapillaris^5^. Despite ongoing cell death, photoreceptors subsequently adapt by modifying their oxygen consumption and metabolism, reducing the rate of cell death^3,6^.

To further understand photoreceptor cell death, and consequently develop new therapies, experimental animal models have been developed. Amongst many experimental animal models of retinal degeneration, the murine retinal detachment model has been widely used given its reproducibility and feasibility to evaluate different cell death pathways, genetic manipulation, or neuroprotective agents^4^. Subretinal injection of sodium hyaluronate 1% is commonly used to detach the neurosensory retina from the RPE^7^. This viscoelastic material induces a retinal detachment of consistent height and duration, in comparison to subretinal saline injection which is prone to reabsorption by the RPE and subsequent RD shallowing or complete resolution^7^. The outcome of the viscoelastic mediated RD is almost exclusive cell death in the photoreceptor layer^6^. As photoreceptor cell death progresses, the ONL cell count and outer nuclear layer (ONL)/inner nuclear layer (INL) thickness ratio are reduced^8,9^. Nonetheless, even with this predictable experimental model, the pattern and rate of photoreceptor cell death is neither uniform nor constant in the detached murine retina^4,10,11^. Despite this being a common knowledge among investigators invested in this field, to date, the regional cell death differences in the detached murine retina have not yet been addressed.

In this work, we investigated the local cell death in a murine experimental retinal detachment model. We analysed the attached and detached retina in different regions and compared the rate of photoreceptor cell death by ONL cell count, ONL/INL ratio, and cell death rate. We found significant regional differences in the detached retina, measured by these three outcome measures. Given this data, we developed three case studies to illustrate common technical pitfalls in cell death analysis in this model. We expect this work will unveil these potential drawbacks and be useful for researchers in this field to detect significant photoreceptor cell death.

## Materials and methods

### Animals

All animals used in experiments and breeding adhered to the statement of the Association for Research in Vision and Ophthalmology (ARVO). Animal protocols were reviewed and approved by the Animal Care Committee of the Massachusetts Eye and Ear Infirmary. All experiments were performed in accordance with institutional regulations. This study was carried out in compliance with the ARRIVE guidelines. C57BL/6J mice were purchased from The Jackson Laboratories (Bar Harbor, ME). Mice were maintained in a standard 12-hour light/dark cycle.

### Retinal detachment model

Eight-week-old male C57BL/6J mice were anesthetized with an intraperitoneal injection of a mixture of 2,2,2-tribromoethanol and 2-methyl-2-butanol at a dose of 125 mg/kg, as previously described^7^. Briefly, a Hamilton injector (Hamilton Company, Reno, NV) with a 34-gauge needle was introduced through a nasal sclerotomy into the subretinal space. Sodium hyaluronate (3.5 µL) was gently injected to detach the neurosensory retina from the underlying RPE. The nasal sclerotomy was sealed with surgical glue. Eyes with haemorrhage, leakage, or cataract were excluded from further analysis. A topical ointment containing bacitracin was applied after the procedure^7^.

### Tissue processing and immunofluorescence

**Figure 1:**
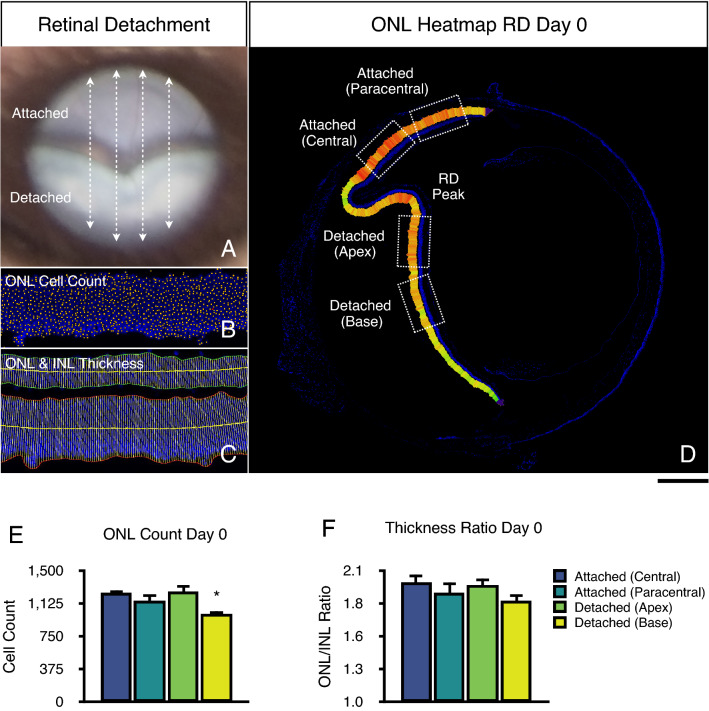
Retinal detachment changes immediately after retinal detachment (baseline, day 0). (**A**) Representative fundus photo of a murine retinal detachment (RD) and sectioning planes (dashed arrows). (**B**) Cryosection image of a 400 µm ONL segment for ONL Cell Count at baseline analysis. (**C**) Cryosection image of a 400 µm ONL segment for outer (ONL) and inner nuclear layer (INL) thickness measurement. (**D**) Representative cryosection whole-retina tile image of a retinal detachment relative ONL thickness heatmap. Regions of interest used for analysis (dotted boxes). (**E**) ONL cell count analysis. (**F**) ONL/INL thickness ratio analysis. Scale bar = 400 µm (**D**). *p < 0.05 vs. attached central region (n = 4–7).

Mice were euthanized at the following timepoints: (1) immediately after detachment (baseline, day 0); (2) day 1; (3) day 7; (4) day 14. Eyes were enucleated immediately after euthanasia and cryopreserved using O.C.T. (Optimal Cutting Temperature, Sakura Finetek, Torrance, CA) at − 20 °C. Cryosections of 10 µm thickness at 1000 µm distance from the first section of the specimen of each sample were cut, mounted, and dried at room temperature (Fig. 1). Slides were frozen at − 80 °C for further processing. Samples were fixed with paraformaldehyde 4% for 30 min. Following two subsequent washes with phosphate-buffered saline, slides were counterstained with 4′,6-diamidine-2′-phenylindole dihydrochloride (Catalogue # D1306, ThermoFisher Scientific, Waltham, MA) for 5 min. Slides were washed with phosphate-buffered saline and mounted with Fluoromount-G Mounting Medium (Catalogue # 00-4958-02, ThermoFisher Scientific, Waltham, MA). Slides were imaged as whole-eye tiles with Zeiss AXIO Imager M2 (Carl Zeiss Inc., Thornwood, NY) fluorescence microscope, and exported in TIFF format for further analysis.

### Local cell death quantitation

The ONL cell count was assessed in an automated manner as previously described by Byun et al.^9^ (Fig. 1B). The ONL and INL thickness was quantitated with the ThicknessTool automated ImageJ plugin using a 1-pixel calliper interval (Fig. 1C)^12^. A retina thickness heatmap was developed as a custom script for the ImageJ platform to display qualitative retinal thickness changes across the detached and attached retina in whole-eye tiles. The relative heatmap color-coded and identified two regions in the detached retina with varying ONL thickness. Two central and paracentral regions in the attached retina were selected for comparative analysis. These four regions of interest with corresponding images from each tile with an ONL length of 400 µm were extracted by a masked observer (Fig. 1D). Retinal regions of interest were defined relative to their distance from the peak of the retinal detachment, as follows: (1) attached central; (2) attached paracentral; (3) detached apex, thus contiguous to the highest point or peak of the RD; and (4) detached base, at a distance of 500 µm from the RD peak. The rate of photoreceptor cell death was estimated by a regression model and expressed as ONL cell count (cells), or ONL/INL ratio (units) change per day. The height of the detachment was measured with a straight calliper as the distance from the RPE to the ONL, perpendicular to each of these cell layers.

### Retinal detachment degeneration criteria

The retinal detachment model causes photoreceptor cell death, which results in ONL cell count reduction and subsequent ONL thinning. Given that the INL is not subject to significant degeneration, the ONL/INL ratio is commonly used to normalize the ONL thickness, given the variations which may occur due to obliquity in tissue sectioning. Taking into account the retinal degeneration that occurs in RD, we proposed a priori the following criteria to determine cell death and degeneration in this model.

1. Absence of significant ONL cell count reduction in the detached retina regions compared to baseline.
2. Absence of significant ONL/INL ratio reduction in the detached retina regions compared to baseline.
3. Progressive ONL cell count reduction in the detached retina through timepoints compared to baseline.
4. Progressive ONL/INL ratio reduction in the detached retina through timepoints compared to baseline.

The ONL cell count and ONL/INL ratio will be assessed in absolute values (a) and normalized to the attached retina (b).

### Statistical analyses

Statistical analysis was performed with SAS software version 11.2.0 (SAS, Cary, NC). Normality was assessed with Shapiro–Wilk test. Statistical significance for differences between groups was determined with one-way ANOVA with Tukey post-hoc correction for multiple comparisons, and T-test for two-group comparisons. Error variance in cell death rate modelling was determined by F-Test. Results are expressed as mean ± standard deviation (SD). A *p* value of < 0.050 was considered statistically significant.

## Results

### Retinal architecture changes immediately after retinal detachment induction

To evaluate the changes in retinal architecture in this model, we first analysed the baseline changes immediately after the induction of a retinal detachment (baseline or day 0). For this purpose, we imaged the entire retina using composite tiles. We then quantitated the ONL thickness in the entire attached and detached retina using an automated retinal thickness measurement plugin for ImageJ^12^. Subsequently, we constructed a retina thickness heatmap using the thickness calliper values. The relative heatmap clearly showed lower ONL thickness values in the detached retina in comparison to the attached retina immediately after detachment (Fig. 1D). Moreover, we observed subtle changes in the heatmap values between two regions in the detached retina, namely the apex and base region of the detached retina. These results suggest that the induction of retinal detachment can subtly modify the outer nuclear layer thickness measured at baseline, before the induction of substantial cell death.

We further assessed this observation by automated ONL cell counting and ONL/INL ratio quantitation. At day 0, the absolute ONL cell count for the central attached retina, paracentral attached retina, detached apex, and detached base was 1247.60 ± 64.62, 1157.80 ± 163.33, 1264.00 ± 150.7, and 1013.80 ± 67.16 cells, respectively (Fig. 1E). There were significant differences between the detached base vs. attached central area (*p* = 0.0335), and between the detached base vs. detached apex area (*p* = 0.0219). Moreover, we normalized the ONL cell counts of the detached retina (RD) to the attached central retina (AR). The normalized RD/AR ONL cell count at baseline was 1.01 ± 0.10 for the detached apex, and 0.81 ± 0.07 for the detached base area. There were significant differences between the detached base vs. attached central (*p* = 0.0020), detached base and attached paracentral (*p* = 0.0.0020); and detached base vs. detached apex (*p* = 0.0011). These results indicate that a significant absolute and normalized ONL cell count reduction can be observed in the detached base region immediately after detachment, before substantial cell death can take place. In addition, only the detached apex region matched criteria 1a and 1b, whereas detached base failed to do so. This reduction in cell count could be consequence of the retina being reshaped, in part due to the viscoelastic's mechanical properties and its delivery to the subretinal space.

The absolute ONL/INL ratio for the central attached retina, paracentral attached retina, detached apex, and detached base at day 0, was 2.00 ± 0.17, 1.92 ± 0.23, 1.98 ± 0.15, and 1.84 ± 0.14, respectively (Fig. 1F). There were no significant differences between any of these regions. The normalized ONL/INL ratio was 0.99 ± 0.13 for the detached apex, and 0.93 ± 0.14 for the detached base area, with no significant differences between any of these regions and the attached retina. These results indicate the ONL/INL was not capable of detecting differences between these detached regions. Thus, both the detached apex and base areas matched criteria 2a and 2b.

Nonetheless, we performed a subgroup analysis of the ONL thickness at baseline. The absolute ONL thickness for the central attached retina, paracentral attached retina, detached apex, and detached base was 94.20 ± 7.08, 85.43 ± 11.78, 88.99 ± 7.42, and 79.96 ± 7.93 µm, respectively. There was a significant difference only between the detached base vs. attached central area (*p* = 0.0268). We calculated the normalized ONL thickness per tile using the attached central retina, as described above. The normalized ONL thickness was 0.94 ± 0.09 for the detached apex, and 0.85 ± 0.08 for the detached base region. Similarly, there was a significant difference only between the detached base vs*.* attached central area (*p* = 0.0056).

Taken together, these results indicate that only detached apex region passed criteria 1 and 2. Moreover, given the significant reduction in the ONL cell count and ONL thickness in the detached base region at baseline, we hypothesize that this detached region may be subject to more mechanical stretching during detachment than the detached apex area, most likely given that the retinal detachment is induced in the peripheral retina close to the *ora serrata*. We speculate that the ONL/INL ratio failed to detect significant differences most likely due to a concurrent ONL and INL stretching.

### Progressive photoreceptor cell death in retinal detachment

**Figure 2:**
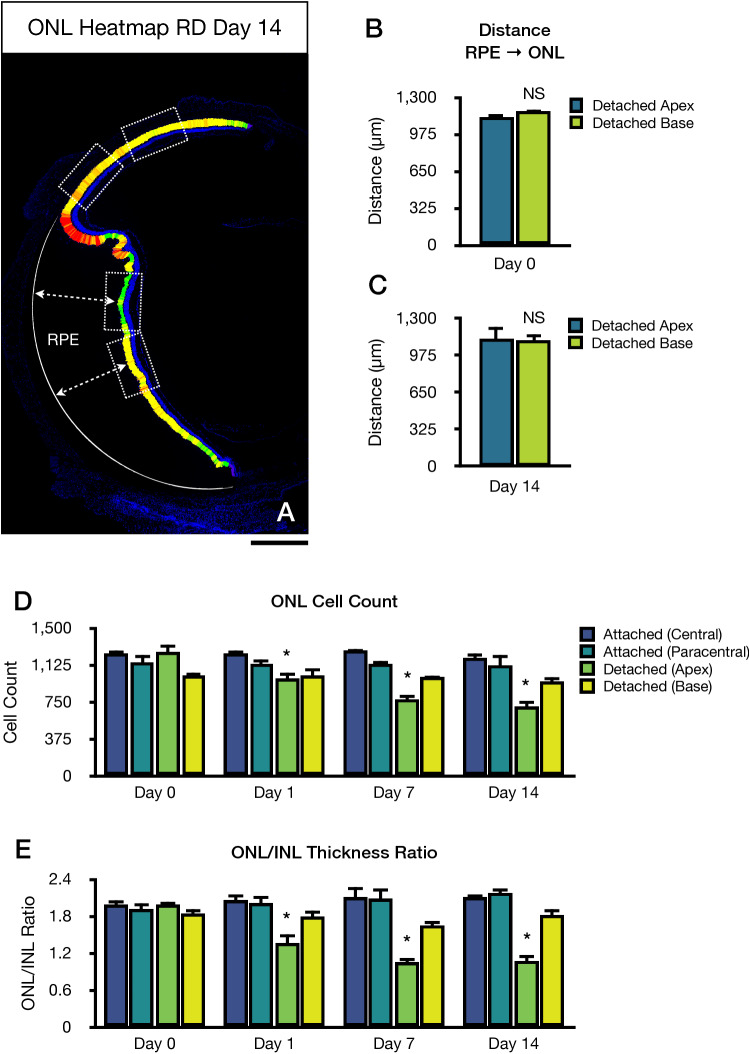
Progressive outer nuclear layer (ONL) degeneration following retinal detachment. (**A**) Representative cryosection whole-retina tile image of a retinal detachment relative ONL thickness heatmap. Regions of interest used for analysis (dotted boxes), and retinal detachment height callipers (dashed arrows). (**B**) Height of retinal detachment at baseline. (**C**) Height of retinal detachment at day 14. (**D**) ONL cell count analysis. (**E**) ONL/INL thickness ratio analysis. Scale bar = 400 µm (**A**). *p < 0.05 vs. baseline day 0 between groups (n = 4–7).

Considering these differences in the detached area, we constructed a relative ONL thickness heatmap 14 days following retinal detachment to better evaluate the degree of retinal degeneration induced by this model. Interestingly, the relative heatmap suggested lower ONL thickness values in the detached apex in comparison to the detached base area (Fig. 2A). Moreover, we observed slight changes between the detached base area compared to both attached areas, even at this timepoint. To further confirm this observation, we analysed the longitudinal changes in both detached apex and detached base areas from day 0 to day 14 (Fig. 2). Of note, no significant changes were observed in the attached retina (central and paracentral) throughout these timepoints compared to baseline. For the detached apex region, the absolute ONL cell count at day 0, 1, 7, and 14 was 1264.00 ± 150.71, 984.40 ± 158.07, 785.80 ± 89.18, 709.20 ± 108.30 cells, respectively (Fig. 2B). There were significant differences between day 0 vs. day 1 (*p* = 0.0171), day 0 vs. day 7 (*p* = 0.0001), and day 1 vs. day 14 (*p* < 0.0001). The ONL/INL ratio in the detached apex region at day 0, 1, 7, and 14 was 1.98 ± 0.16, 1.36 ± 0.31, 1.05 ± 0.20, and 1.08 ± 0.23, respectively (Fig. 2C). There were significant differences between day 0 vs. day 1 (*p* = 0.0006), day 0 vs. day 7 (*p* < 0.0001), and day 0 vs. day 14 (*p* < 0.0001). These results indicate that the detached apex area displays progressive cell count reduction from day 0 to 14. Thus, this area fulfilled criteria 3 and 4, and therefore it is appropriate to determine progressive retinal degeneration in this model.

For the detached base area, the absolute ONL cell count at day 0, 1, 7, and 14 was 1013.80 ± 67.16, 1017.60 ± 163.62, 1001.00 ± 27.93, and 954.00 ± 122.38, respectively (Fig. 2B). There were no significant differences between day 0 and any later timepoint in this detached region. The ONL/INL ratio in the detached base region at day 0, 1, 7, and 14 was 1.85 ± 0.15, 1.79 ± 0.23, 1.66 ± 0.15, 1.81 ± 0.24, respectively (Fig. 2C). There were no significant differences between day 0 and any later timepoint in this detached region. These results suggest that the detached base area did not pass criteria 3 and 4, therefore was unfit to determine progressive retinal degeneration in this retinal detachment model.

Finally, given the aspect of the detached bullous retina, we hypothesized that the observed differences in ONL count and ONL/INL ratio between the detached apex and detached base were not entirely dependent on the height of the retinal detachment. At baseline, the distance from the RPE to the ONL, or height of retinal detachment, was 1126.75 ± 62.02 µm for the detached apex and 1171.03 ± 48.14 µm for the detached base. On day 14, the height of retinal detachment was 1120.77 ± 223.85 µm for the detached apex and 1121.77 ± 144.17 µm for the detached base. There were no statistically significant differences in the height of retinal detachment between the detached apex and detached based at baseline (*p *= 0.244) or day 14 (*p *= 0.993). Collectively, these results suggest that the observed local differences in photoreceptor cell death are not entirely due to the height of the retinal detachment, which may suggest different adaptive mechanisms in these regions. Moreover, there were no statistically significant differences in the height of retinal detachment from baseline compared to day 14 in the detached apex (*p *= 0.956) or detached base (*p *= 0.552). These results further demonstrate that the retinal detachment achieved with this model is stable and reproducible.

### Photoreceptor cell death rate in retinal detachment

**Figure 3:**
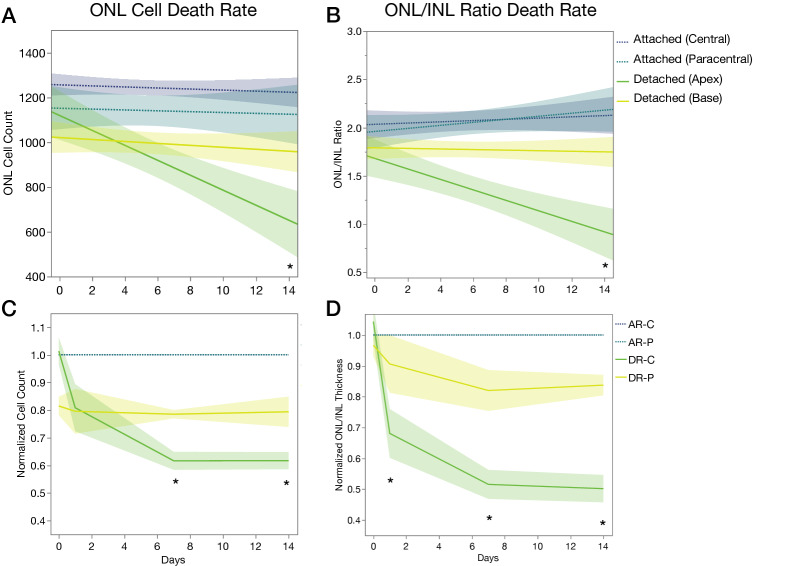
Cell death rate and normalized cell death following retinal detachment. (**A**) ONL cell death rate. (**B**) ONL/INL thickness ratio cell death rate. (**C**) Case 1: normalized ONL cell count. (**D**) Case 1: normalized ONL/INL thickness ratio. *p < 0.05 (n = 4–7).

Next, we calculated the rate of photoreceptor cell death per day in all regions by ONL cell count and ONL/INL ratio (Fig. 3A,B). The cell death rate by ONL cell count for the central attached retina, paracentral attached retina, detached apex, and detached base region was − 2.34 (*p* = 0.4353), − 1.90 (*p* = 0.7482), − 33.40 (*p* < 0.0001), and − 4.35 (*p* = 0.3020), respectively (Fig. 3A). The cell death rate by ONL/INL ratio for the central attached retina, paracentral attached retina, detached apex, and detached base region was 0.01 (*p* = 0.4701), 0.01 (*p* = 0.1491), − 0.05 (*p* = 0.0002), and − 0.01 (*p* = 0.6808), respectively (Fig. 3B). Collectively, these results suggest that only the detached apex region had a significant and progressive cell death rate compared to baseline, with an estimate of 33.40 photoreceptors dying per day and a 0.05 reduction of ONL/INL ratio per day.

### Case studies

In summary, the detached apex outperformed the detached base area in detecting expected and significant differences between the attached and detached retina by means of common outcome variables in retinal degeneration models such as ONL cell count and ONL/INL ratio. In addition, the detached base region appears to be subject to a more pronounced mechanical stretching during the induction of the retinal detachment. Moreover, this is further compromised by a lower rate in ONL cell count and ONL/INL ratio reduction. Given these significant findings, we propose three case studies to explore different methods and emulate several researchers’ approach in this model. We will assess the capacity of the chosen variables and group comparisons to detect true and significant differences in retinal detachment.

#### Case 1: normalized ONL count

We will assume that the investigator, in this case will evaluate the detached retina at different timepoints compared to day 0. For this purpose, the investigator will compare the ONL cell count and ONL/INL ratio in the detached apex or base as a ratio of the attached retina per timepoint. We will use the attached central retina area for normalization purposes (Fig. 3C,D).

If the investigator uses the detached apex area, the normalized ONL cell count at day 0, 1, 7, and 14 will be 1.01 ± 0.10, 0.80 ± 0.17, 0.61 ± 0.07, and 0.61 ± 0.06, respectively (Fig. 3C). There will be significant differences between day 0 vs. day 7 (*p* = 0.0003), day 0 vs. day 14 (*p* = 0.0005). If the ONL/INL ratio is expressed as a function of the attached retina, this normalized ONL/INL ratio at day 0, 1, 7, and 14 will be 0.99 ± 0.13, 0.65 ± 0.13, 0.50 ± 0.13, and 0.51 ± 0.13, respectively (Fig. 3D). There will be significant differences between day 0 vs. day 1 (*p* = 0.0008), day 0 vs. day 7 (*p* < 0.0001), day 0 vs. day 14 (*p* < 0.0001).

However, if the investigator uses the detached base area, the normalized ONL cell count at day 0, 1, 7, and 14 will be 0.81 ± 0.07, 0.79 ± 0.16, 0.78 ± 0.03, and 0.79 ± 0.10 respectively (Fig. 3C). There will be no significant differences between day 0 and any later timepoint in this detached region. The normalized ONL/INL ratio at day 0, 1, 7, and 14 will be 0.93 ± 0.14, 0.87 ± 0.15, 0.80 ± 0.14, and 0.86 ± 0.11, respectively (Fig. 3D). There will be no significant differences between day 0 and any later timepoint in this detached region.

In summary, if the investigator chooses the detached base region, it will fail to demonstrate significant true differences between the detached and attached retina. In essence, it will fail to reject of a false null hypothesis, thus falling into a type II error.

#### Case 2: two-group comparison

**Figure 4:**
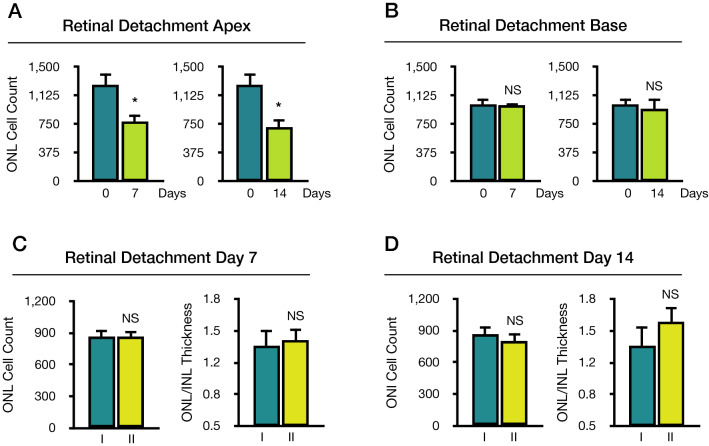
Cases 2 and 3. Case 2: Two-group outer nuclear layer (ONL) cell count comparison between baseline and day 7 or 14 following retinal detachment. (**A**) Retinal detachment apex area. (**B**) Retinal detachment base area. Case 3: Alternate use of apex and base region in retinal detachment. Images from detached apex and base areas were pooled and randomly shuffled to two groups, I and II. (**C**) Comparison between groups I and II at baseline and day 7. (**D**) Comparison between groups I and II at baseline and day 14. *p < 0.05 vs. baseline, *NS* not significant vs. baseline (n = 5–7).

Another investigator in this case, will try to shortcut his experiment by comparing only two timepoints. It will compare the detached retina at day 0 vs day 7 or 14, using a two-group mean statistical comparison test (Fig. 4A,B).

If the investigator uses the detached apex area (Fig. 4A), the ONL cell count will demonstrate significant differences between day 0 vs. day 7 (*p* < 0.0001), day 0 vs. day 14 (*p* < 0.0001). The ONL/INL ratio will demonstrate similar significant differences between day 0 vs. day 7 (*p* < 0.0001), day 0 vs. day 14 (*p* < 0.0001). If the investigator uses the detached base area (Fig. 4B), the ONL cell count will fail to demonstrate significant differences between day 0 vs. day 7 (*p* < 0.8543), day 0 vs. day 14 (*p* < 0.3962). Moreover, the ONL/INL ratio will also fail to demonstrate significant differences between day 0 vs. day 7 (*p* < 0.2820), day 0 vs. day 14 (*p* < 0.9902). In summary, a two-tailed two-group comparison will also fail to demonstrate any significant differences in the detached base region.

#### Case 3: alternate use of detached apex or base areas

Finally, the investigator in this case, will compare the effect of treatment compared to a control, on day 7 or 14. However, the researcher is not aware of the regional cell death differences in the detached retina between the detached apex and base areas; therefore, it will acquire images from the detached retina at these regions indistinctively. To simulate this selection, we pooled detached apex and detached base images from day 7 or 14 and randomly allocated them to two groups labelled I for the control group, and II for the treatment group (Fig. 4C,D).

On day 7, the ONL cell count for groups I and II was 867.83 ± 146.98 and 859.50 ± 151.73 cells, respectively (Fig. 4C). No significant differences were observed between group I and II (*p* = 0.9249, T-test). The ONL/INL ratio for the group I was 1.32 ± 0.43 and 1.38 ± 0.30 for group II. Similarly, no significant differences were observed between groups I and II (*p* = 0.7906, T-test). On day 14, the ONL cell count for groups I and II was 864.60 ± 175.50 and 798.60 ± 175.03 cells, respectively (Fig. 4D). No significant differences were observed between groups I and II (*p* = 0.5680, T-test). The ONL/INL ratio for the group I was 1.32 ± 0.49 and 1.57 ± 0.38 for group IΙ. Similarly, no significant differences were observed between groups I and II (*p* = 0.3593).

These results suggest that lack of consistency in RD region selection for further analysis can cancel previously observed significant differences between these two regions on day 7 in the ONL cell count (*p* = 0.0033) and ONL/INL ratio (*p* < 0.0001). Similarly, differences at day 14 in the ONL cell count (*p* = 0.0103) and ONL/INL ratio (*p* = 0.0003) would also be masqueraded. In summary, if the investigator neglects the regional cell death differences in this model, it will fail to reject a false null hypothesis.

## Discussion

In this study, we analysed the morphological changes in the retina after detachment. We evaluated the ONL cell count and ONL/INL ratio in the entire retina at baseline and observed significant morphological changes between RD regions immediately after detachment. Moreover, we identified two regions in the detached retina subject to different cell death rates. We speculate that mechanical and regional differences in the bullous detached retina can modify the rate of cell death in this model.

The murine retinal detachment model has been widely established to study cell death mechanisms in the retina^6,7,13^. As sodium hyaluronate is injected into the subretinal space close to the *ora serrata*, it detaches the retina from the periphery towards the equator and posterior pole. As the bullous RD force vector moves posteriorly towards the central retina, it is expected that a certain degree of dragging and stretching of this initial peripheral detached region will occur to accommodate this added subretinal volume (~ 3 to 4 µL). In addition, once the detached retina is in apposition with the large murine lens, this latter structure may act as a partially opposing vector to the bullous detachment vector. Hence, part of the detachment vector can be neutralized by this lens vector, at the expense of compacting the retinal tissues. However, the remainder unopposed detachment vector contributes to stretching, which is more evident in the detached base than detached apex, probably given the more central location of the latter region. Finally, we speculate that the lesser amount of stretching at the detached apex area and apex itself could be due to an already attenuated RD force vector in the central detached retina given the injected volume, and probably a different magnitude and direction of the lens vector given the smaller radius of contact between these two structures at the apex.

Given the different cell death kinetics of cones and rods in retinal degeneration^14^, their response to retinal detachment can vary, in context with the various rod/cone ratio across species. Interestingly, Linberg et al*.* have previously demonstrated, in a cone-dominant ground squirrel experimental retinal detachment, a similar photoreceptor cell death percentage across three different detached regions^15^. In the ground squirrel, the overall photoreceptor population is composed of a majority of cones (86%) and a few remaining rods (14%)^15,16^. In our work, we used the C57BL/6J mice, which has a ratio of 34 rods to 1 cone at an eccentricity of 20° for the optic nerve, similar to 30 rods to 1 cone in humans^17^. We believe these similarities in the rod/cone ratio can further support the applicability of this experimental model.

The height of retinal detachment has been posed as a critical variable in cell death induction in this injury. Increased distance of photoreceptors to the RPE and choriocapillaris, and thus less availability of nutrients and potentially oxygen, is the most plausible mechanism to trigger cell death pathways. As the retina is detached, the presence of a subretinal space increases the latency of oxygen delivery to the photoreceptors from the choriocapillaris^5^. However, as the choriocapillaris flows continue to be uninterrupted, we speculate that diffusion into the subretinal space should be maintained. Therefore, the initial oxygen partial pressure should eventually reach an equilibrium with the plasma oxygen partial pressure. However, the time to equilibrium is critical and probably not prompt enough to provide adequate metabolic support to photoreceptors initially. Furthermore, oxygen consumption by photoreceptors maybe altered and infiltrating subretinal immune cells may also consume part of the oxygen originally destined for photoreceptors. Still, given the fact that cell death kinetics slow down over time^10^ and photoreceptor cell loss is observed to reach a plateau, we hypothesize that a combination of moderate O_2_ and nutrient supply and other local factors can contribute to rescue the retina in this injury model. Such a premise is insufficient to address the differences between different regions in the retina. It remains elusive why photoreceptors in the detached base display a different cell death as compared to the detached apex area. We can speculate about the role of mechanical factors for these disparities. However, there may be other regional factors related to oxygen consumption and metabolic adaptation on the retina, which may be further affected by the local densities of rods/cones or glial cells, or even unknown factors.

There are several limitations to this study. First, we used axial cryosections to evaluate the morphology of the attached retina. We believe it is necessary to have a detached and attached region in the same slide, to obtain a normalized ratio if needed, as described above. However, investigators have various ways of processing the enucleated eyes. While some perform sagittal or coronal cryosections, axial cryosections allow us to evaluate the true height of the detachment, otherwise not feasible by other methods, as these former two sectioning strategies do not necessarily have the attached retina in the same section. Second, we use a cropped section of 400 µm to assess cell death. Though a larger area may seem more appropriate, regional differences between detached apex and detached base areas may mask subtle photoreceptor cell count reduction by merging these two regions, which present different cell death rate. Finally, regarding the area between the retinal detachment apex and the attached retina, we refrained from using this region due to the following concerns: (1) the area or region of interest in this initial portion of the detached retina was often irregular and did not always measure at least 400 µm, and thus did have smaller area values which could induce another source of bias; (2) investigators are prone to analyse photoreceptor survival and outcomes in the detached retina, usually in contact with the lens; (3) a critical factor in the differences between the detached apex and detached base is that both areas are in contact with the lens, and most importantly equidistant and parallel to the RPE, which argues towards comparable oxygen and nutrient delivery to these areas from the choriocapillaris. The area between the attached retina and detached apex is perpendicular to the RPE, and may receive different oxygen and nutrient delivery across its area.

In summary, baseline changes can be observed in the retina immediately after detachment. Moreover, cell death rates differ across regions in the detached retina, most likely given a combination of mechanical and metabolic adaptive changes in the photoreceptor layer. We encourage the use of axial cryosections with a detached and attached retina in the same section to normalize their respective ONL cell count and ONL/INL ratio. In addition, cell death analysis performed in the detached apex region can detect true retinal degeneration. We believe this systematic approach to evaluating photoreceptor cell death can help reduce biases and advance knowledge in photoreceptor cell death in retinal disease models.

## Literature search

The biomedical literature from MEDLINE was searched through PubMed on March 20, 2020. The following terms were used: *retinal detachment, photoreceptor, cell death, mouse,* and *regional difference*. Only results in the English language were selected for further analysis.

## References

[1] Fisher SK, Lewis GP, Linberg KA, Verardo MR (2005). Cellular remodeling in mammalian retina: Results from studies of experimental retinal detachment. Prog. Retin. Eye Res..

[2] Murakami Y (2013). Photoreceptor cell death and rescue in retinal detachment and degenerations. Prog. Retin. Eye Res..

[3] Stone J (1999). Mechanisms of photoreceptor death and survival in mammalian retina. Prog. Retin. Eye Res..

[4] Trichonas G (2010). Receptor interacting protein kinases mediate retinal detachment-induced photoreceptor necrosis and compensate for inhibition of apoptosis. Proc. Natl. Acad. Sci. USA.

[5] Wang S, Linsenmeier RA (2007). Hyperoxia improves oxygen consumption in the detached feline retina. Investig. Ophthalmol. Vis. Sci..

[6] Cook B, Lewis GP, Fisher SK, Adler R (1995). Apoptotic photoreceptor degeneration in experimental retinal detachment. Invest. Ophthalmol. Vis. Sci..

[7] Matsumoto H, Miller JW, Vavvas DG (2013). Retinal detachment model in rodents by subretinal injection of sodium hyaluronate. J. Vis. Exp..

[8] Byun, J. *et al.* Quantifying structural distortions in retinal tissue before and after injury. In *Workshop on Multiscale Biological Imaging, Data Mining & Informatics, Santa Barbara, CA, USA* 10–11 (2006).

[9] Byun J (2006). Automated tool for the detection of cell nuclei in digital microscopic images: Application to retinal images. Mol. Vis..

[10] Matsumoto H (2014). Strain difference in photoreceptor cell death after retinal detachment in mice. Invest. Ophthalmol. Vis. Sci..

[11] Kayama M (2011). Heat shock protein 70 (HSP70) is critical for the photoreceptor stress response after retinal detachment via modulating anti-apoptotic Akt kinase. Am. J. Pathol..

[12] Maidana, D. E. *et al.* ThicknessTool: automated ImageJ retinal layer thickness and profile in digital images. *Sci. Rep.* **10**, (2020). 10.1038/s41598-020-75501-y PMC7595229 33116161

[13] Anderson DH, Guerin CJ, Erickson PA, Stern WH, Fisher SK (1986). Morphological recovery in the reattached retina. Investig. Ophthalmol. Vis. Sci..

[14] Murakami Y (2012). Receptor interacting protein kinase mediates necrotic cone but not rod cell death in a mouse model of inherited degeneration. Proc. Natl. Acad. Sci. USA.

[15] Linberg KA, Sakai T, Lewis GP, Fisher SK (2002). Experimental retinal detachment in the cone-dominant ground squirrel retina: Morphology and basic immunocytochemistry. Vis. Neurosci..

[16] Kryger Z, Galli-Resta L, Jacobs GH, Reese BE (1998). The topography of rod and cone photoreceptors in the retina of the ground squirrel. Vis. Neurosci..

[17] Volland S, Esteve-Rudd J, Hoo J, Yee C, Williams DS (2015). A comparison of some organizational characteristics of the mouse central retina and the human macula. PLoS ONE.

